# Plan U: Universal access to scientific and medical research via funder preprint mandates

**DOI:** 10.1371/journal.pbio.3000273

**Published:** 2019-06-04

**Authors:** Richard Sever, Michael Eisen, John Inglis

**Affiliations:** 1 Cold Spring Harbor Laboratory, Cold Spring Harbor, New York, United States of America; 2 University of California, Berkeley, California, United States of America; 3 Howard Hughes Medical Institute, Chevy Chase, Maryland, United States of America

## Abstract

Preprint servers such as arXiv and bioRxiv represent a highly successful and relatively low cost mechanism for providing free access to research findings. By decoupling the dissemination of manuscripts from the much slower process of evaluation and certification by journals, preprints also significantly accelerate the pace of research itself by allowing other researchers to begin building on new results immediately. If all funding agencies were to mandate posting of preprints by grantees—an approach we term Plan U (for “universal”)—free access to the world’s scientific output for everyone would be achieved with minimal effort. Moreover, the existence of all articles as preprints would create a fertile environment for experimentation with new peer review and research evaluation initiatives, which would benefit from a reduced barrier to entry because hosting and archiving costs were already covered.

## Abstract 2

Preprint servers are a low-cost mechanism for providing free access to research findings, and can also significantly accelerate research itself by making results available immediately. This Perspective article proposes that funding agencies should mandate preprint posting to ensure universal free access to the world’s scientific output, as well as stimulate new peer review and research evaluation initiatives.

## Introduction

Since its inception, the Internet has had the potential to provide free and rapid access to the results of scientific and medical research. However, despite numerous efforts, this potential remains largely unrealized. The incumbent system of research communication has 2 main problems. It is slow: It typically takes around a year for a manuscript submitted to a research journal to be peer-reviewed, accepted, and published online [1]. And research outputs are not widely accessible: Many papers are published in journals available exclusively via subscription, which may restrict their readership to researchers in certain universities and countries.

Previous attempts to address these problems have focused on changing the economics of journal publishing, shifting from subscription journals to publications funded by upfront payments that enable immediate free access to published articles, or on encouraging governments and funders to mandate the deposition of published papers in repositories after an embargo period in which journals continue to charge subscriptions for immediate access.

These efforts have made a growing proportion of the research literature freely and immediately available online, but researchers complain that overall delays to publication are getting longer, and most published papers remain behind paywalls during the period they are of most value in accelerating research [2, 3].

## Providing free access via preprint servers

Arguably the most effective mechanism for providing free, immediate access to research has been the nonprofit preprint server arXiv [4]. In operation for 28 years, arXiv provides free access to nearly 1.5 million papers in the mathematical and physical sciences, with 140,000 additions each year. Its success has inspired over the past 5 years the launch of numerous discipline-specific servers, including bioRxiv, chemRxiv, and EarthArxiv. Not only do these servers provide immediate, universal access to all papers but they also provide this access far earlier than journals, because preprints are typically posted prior to or coincident with journal submission.

The growing popularity of preprints speaks to the desire of authors to provide early evidence of productivity and share their work with colleagues as soon as they choose to do so, together with their frustration with the slow pace of the journal publication process. The attention being given to preprints by readers highlights the importance they place on receiving and evaluating newly available information.

The early availability of new research on preprint servers allows other researchers, where appropriate, to begin building on the results immediately; a rough estimate is that the aggregate time saving could make the pace of scientific discovery 5 times faster over 10 years [5]. Preprint posting can also help authors improve manuscripts by enabling them to receive public and private feedback on their work from a much larger number of individuals than during traditional peer review. Community response may not only improve manuscripts in development but also increase the efficiency and effectiveness of subsequent peer review by addressing inadequacies upstream.

Because preprint servers do not perform peer review (see below), they are able to operate at low per-paper costs that can be covered via central funding, making them free at the point of use to both authors and readers. With such low per-paper costs, the world′s entire research output could be accommodated on preprint servers relatively easily.

## A preprint mandate

If all research funders required their grantees to post their manuscripts first on preprint servers—an approach we refer to as Plan U (for “universal”)—the widespread desire to provide immediate free access to the world’s scientific output would be achieved with minimal effort and expense. As noted above, mathematicians, physicists, and computer scientists have been relying on arXiv as their primary means of communication for decades. The biomedical sciences were slower to adopt preprinting, but bioRxiv is undergoing exponential growth and several million readers access articles on bioRxiv every month [6]. Depositing preprints is thus increasingly common among scientists and mandating it would simply accelerate adoption of a process many predict will become universal in the near future.

There is a precedent for mandating preprint deposition: since 2017, the Chan Zuckerberg Initiative (CZI) has mandated that all grantees deposit preprints prior to or at submission for formal publication [7]. This requirement has been accepted by CZI-funded investigators, many of whom were already routinely depositing manuscripts on bioRxiv.

## Peer review

Plan U would establish preprint servers as the de facto means for disseminating all scientific research, which has long been the case in fields covered by arXiv. It is assumed that most preprints would subsequently be peer reviewed. This could occur via variations of the current system, with author submission to journals, reviews, revisions, and decisions to accept or reject proceeding as they currently do but with a vital difference: The work in question would be available to interested readers while these processes take place. This is more or less what happens in physics today, with arXiv providing access and journals providing peer review.

The availability and permanent online archiving of manuscripts before their evaluation would also provide an opportunity for innovation in how peer review is organized and performed and how it might be tailored to the needs of particular disciplines and audiences. Crucially, because the costs of ingestion, online display and permanent archiving of manuscripts would already be covered by a preprint server, there is a reduced barrier to entry for new peer review initiatives that emphasize curation, commentary, and evaluation rather than manuscript hosting.

Plan U therefore creates fertile ground for a dynamic new ecosystem, opening opportunities for experimentation with peer review rather than prescribing a particular process, endpoint, or business model. Such flexibility may be of particular benefit to scientific societies, nonprofit organizations, journals, and self-organizing groups of academics who wish to improve on existing approaches to peer review and/or explore alternative ways to evaluate academic output.

## Preprint server and posting requirements

When fully implemented, Plan U would define minimal requirements for preprint servers. These would include permanence of deposition (articles cannot be removed but may be flagged as withdrawn for a specific reason), the ability to revise an article, easily indexable and standardized metadata (title, author, abstract, unique article identifiers [IDs], uniform resource identifers [URIs], etc.), provisions for text mining, forward linking to evaluation and/or certification mechanisms (e.g., traditional journals, overlay platforms, and badging agencies), and guaranteed long-term preservation strategies (e.g., deposition in dedicated dark archives such as Portico or CLOCKSS). Under Plan U, funders would mandate that grantees post preprints as early as possible; however, they could allow posting delays for research linked to patent filing, for which a preprint, like other forms of dissemination, would constitute public disclosure.

## Relationship to other public access mandates

Plan U advances the goals of efforts by funders to achieve free access to the research they sponsor, such as the United States Office of Science and Technology Policy (OSTP) Public Access policy [8], the National Institutes of Health (NIH) mandate [9], and the recent Plan S proposal [10]. But because it sidesteps the complexities and uncertainties of attempting to manipulate the economics of a US$10B/year industry, Plan U could literally be mandated by funders tomorrow with minimal expense, achieving immediate free access to research and the significant benefits to the academic community and public this entails. Funders and other stakeholders could then focus their investment and innovation energies on the critical task of building and supporting robust and effective systems of peer review and research evaluation.

## Abbreviations

CZI: Chan Zuckerberg Initiative
ID: identifier
NIH: National Institutes of Health
OSTP: Office of Science and Technology Policy
URI: uniform resource identifier
